# An Integrative Appraisal Model of Epistemic Curiosity

**DOI:** 10.1007/s42761-025-00328-7

**Published:** 2025-11-13

**Authors:** Asli Erdemli, Catherine Audrin, David Sander

**Affiliations:** 1 Swiss Center for Affective Sciences (CISA), University of Geneva, State of Geneva, Geneva, Switzerland; 2 Department of Psychology, FPSE, University of Geneva, State of Geneva, Geneva, Switzerland; 3 University of Teacher Education, Vaud, Switzerland

**Keywords:** Curiosity, Reward, Appraisal, Wanting, Anticipation, Knowledge

## Abstract

Epistemic curiosity is an affective state characterized by the desire for knowledge. This review paper suggests an integrative appraisal model of epistemic curiosity, which combines information-gap, emotion and reward-anticipation accounts of curiosity while proposing an appraisal theory-based explanation for why some knowledge gaps elicit curiosity. We discuss evidence for the involvement of brain circuits that support each step of curiosity-driven knowledge exploration. Overall, this integrative appraisal model of curiosity suggests that epistemic curiosity i) is an emotion which is triggered by appraised high relevance, valence, novelty and coping potential of knowledge-gaps; ii) confers a reward value to its epistemic object; iii) triggers a reward anticipation process; iv) and motivates a knowledge-seeking behavior (i.e., “wanting knowledge”). Once curiosity is satisfied, future knowledge-seeking expectations are updated according to affective prediction errors (i.e., “liking and learning”). If relevance is appraised as high but coping potential is appraised as low, the knowledge-gap rather creates anxiety and leads to information-avoidance. Future directions as to how this integrative model can be tested and extended are also discussed.

Healthy adults spend a lot of time, resources, and effort in pursuit of knowledge. Endeavors to seek knowledge range from simple actions such as reading the newspaper in the morning to life-long dedications such as academic research. Clearly, *Homo sapiens* is motivated to *know*. Currently, multiple theories coexist in scientific literature to explain why and what humans are interested in knowing: information-gap, emotion, reward-anticipation and computational accounts of curiosity offer each seemingly -at least partially- incompatible perspectives on the subject, originating from—and perhaps unintentionally reinforcing—separate approaches of the same phenomenon. The aim of this paper is to propose a unified framework for curiosity through an appraisal perspective. First, we provide a brief overview of the main modern theoretical accounts of epistemic curiosity. Second, we present the integration of these models, with a particular emphasis on the reward-based framework, with an appraisal-based model of knowledge-seeking and discuss how this new model is able to account for a large variety of findings in curiosity research, some of which so far unexplained by previous theories. In particular, the integrative appraisal model of curiosity formulates testable hypotheses about why not every knowledge gap elicits curiosity. Finally, we discuss future directions which can be taken to test the model and matters which require clarification.

Within the scope of this article, we will refer to curiosity as wanting non-instrumental knowledge (i.e., “knowledge for knowledge” rather than knowledge for the facilitation of goal achievement, see also Sharot & Sunstein, 2020; Shin & Kim, 2019; Szumowska & Kruglanski, 2020), or put differently, to “the desire for information in the absence of extrinsic reward” (Markey & Loewenstein, 2014, p.228). Whether curiosity and interest refer to the identical psychological process or not is a matter of great debate (see Ainley, 2019; Grossnickle, 2016; Pekrun, 2019; Schmidt & Rotgans, 2021; Tang et al., 2022). Here, we use interest to refer to the enjoyment and engagement felt about the desired knowledge once it is obtained (Shin & Kim, 2019). In this paper, we focus mainly on *state* curiosity as an affective *episode*; however, curiosity can also be studied in trait form, which can be defined as an “individual’s personality disposition toward experiencing curiosity” (Markey & Loewenstein, 2014, p. 230; see also Wagstaff et al., 2020 for a review on trait curiosity).

During the past century, the curiosity-driven pursuit of knowledge has given birth to many theories (see Berlyne, 1950, for a review of theories prior to 1950). Among the most notable theories, we will briefly synthesize three modern accounts of curiosity which currently coexist in literature: the information-gap account, the emotion-account, and the reward-anticipation account.

## Theories of Curiosity

### Information-gap Account of Curiosity

The information-gap theory proposed by Loewenstein (1994) is inspired by both drive and uncertainty reduction theories, and posits that epistemic curiosity emerges when a gap between a person’s existing and desired level of knowledge on a specific topic captures their attention (Golman & Loewenstein, 2018; Loewenstein, 1994; Markey & Loewenstein, 2014). In such a theoretical perspective, curiosity is conceptualized as a feeling of deprivation (Loewenstein, 1994), even more so when not satisfied (Markey & Loewenstein, 2014). In support of this theory, Jepma et al., (2012) found that perceptual curiosity, induced by blurry images, activated brain regions associated with conflict and arousal (i.e., anterior insula and anterior cingulate cortex) whereas the relief of curiosity activated brain regions associated with reward processes (e.g., striatum and orbitofrontal cortex). By viewing curiosity as an aversive experience (Markey & Loewenstein, 2014), the information-gap theory of curiosity is typically conceived as the opposite of the reward-anticipation theory of curiosity.

Although many researchers do not necessarily agree with curiosity being an aversive state, the knowledge gap concept (i.e., the difference between desired and actual knowledge) has inspired research that followed.

### The Epistemic Emotion Account of Curiosity

In contrast to the information-gap theory’s drive-like approach to curiosity, the emotion account of curiosity conceptualizes curiosity as an emotion (see Nerantzaki et al., 2021), i.e., a fast, multicomponentional affective phenomenon composed of two distinct phases: elicitation and response (Sander, 2013). Appraisal theories of emotion suggest that emotion is elicited as a result of automatic evaluations of the environment (see Scherer & Moors, 2019, for a review on appraisal theories). Once triggered, the emotional response is physiological (e.g., heart rate, perspiration, pupil dilation, respiration rate), motor (e.g., facial, vocal, and bodily expression), motivational (i.e., action tendencies), and consciously experienced (i.e., feeling; see Sander et al., 2018). Although curiosity’s status as an emotion is not universally acknowledged, research suggests that curiosity has a corresponding subjective feeling (Tang et al., 2022), cognitive appraisal profile (see Noordewier & Goclowksa, 2024), bodily expression (for curiosity: Lyu et al., 2022; for interest: Dukes et al., 2017), physiological changes (Kang et al., 2009), and motivational tendencies towards exploration (Poli et al., 2024; Vogl et al., 2019).

**Fig. 1:**
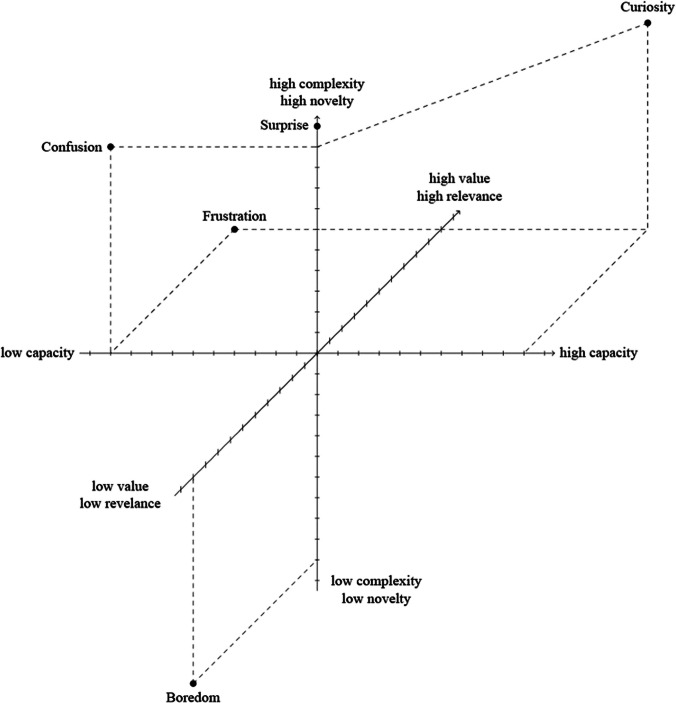
An illustration of curiosity as an emotion in 3D appraisal-space. *Note*: This figure illustrates how curiosity is predicted by appraisals of novelty/complexity, capacity (i.e., coping potential), and value (e.g., goal-relevance or importance), according to the emotion account of curiosity. It also represents curiosity in relation to other epistemic emotions in a 3D appraisal space. This illustration is inspired by the 2D appraisal space representations of confusion and interest in (Silvia, 2010), and confusion and curiosity in Muis et al., (2018) which also represents how confusion might evolve into frustration or boredom as appraisals change during an epistemic task

Some of the cornerstone research on interest as an emotion are the studies of Silvia (2005, 2008, 2010) which use the appraisal theories of emotion to identify appraisal profile of *interest*. Silvia’s work was inspired by the “collative variables” (i.e., variables describing the stimuli obtained through comparison of the stimulus with other stimuli or with itself across time, Berlyne, 1966, p. 30) used by Berlyne to predict *curiosity* and, more specifically, made use of the component process model developed by Scherer (see Scherer, 2009). Results suggested that interest is predicted by appraisals of novelty, complexity and coping potential, meaning that if a stimulus was appraised as novel, complex yet also understandable, it triggered one’s interest (Silvia, 2005; see Audrin & Coppin, 2022). Further research suggested that personal value or relevance indicating factors such as goal-relevance (Audrin & Coppin, 2022; Connelly, 2011) and social or personal relevance (Dubey et al., 2022) are important ingredients of interest/curiosity (see Fig. 1).

Curiosity is often studied under the epistemic emotion taxonomy (see the Epistemically-Related Emotion Scale in Pekrun et al., 2017). Epistemic emotions refer to the “emotions that arise when the object of their focus is knowledge and knowing” (Muis et al., 2015, p.173). Epistemic emotion researchers investigated curiosity’s antecedents (e.g., the appraisals of perceived value, complexity, novelty, capacity, goal congruence, see Muis et al., 2018) as well as its consequences (e.g., higher knowledge exploration, Vogl et al., 2020). The research on epistemic curiosity as an emotion closely overlapped with the construct of “situational interest” in educational psychology (see Hidi and Reninger, 2006 for the four-phase interest development model).

Epistemic emotion research also drew attention to the dynamic of affective states during one’s cognitive engagement with knowledge: surprise might lead to curiosity or confusion, which might lead to enjoyment or frustration or boredom as an individual is confronted with new knowledge (D’Mello & Graesser, 2012; Muis et al., 2018; Fig. 1).

### The Reward-Anticipation Account of Curiosity

**Fig. 2:**
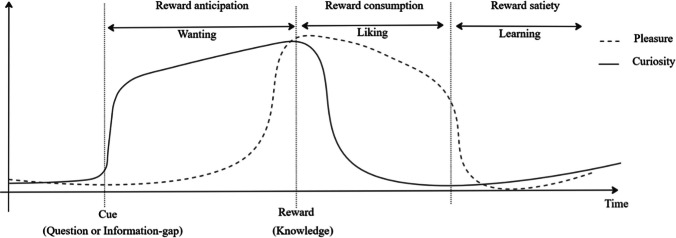
The reward cycle of curiosity-eliciting knowledge: curiosity as epistemic reward-anticipation. *Note*: Adapted from Berridge and Kringelbach (2011), this figure illustrates the reward cycle of (curiosity eliciting) knowledge: The dashed line represents how pleasure arises right before reward consumption, lasts during reward consumption and wanes as the individual experiences satiety. The innovative parts of this figure are the application of this model to knowledge, and the full line representing curiosity’s evolution across time: according to the reward anticipation account of curiosity, curiosity is the wanting of (interesting) knowledge. Curiosity is aroused with a reward cue, which in this case is for instance a question (e.g., “What is the most popular album, ever?”) or the attentional focus on an information-gap (e.g., “I know barely anything about my long-time neighbor.”). Curiosity grows as one gets closer to the wanted knowledge and wanes once it is obtained. The individual might feel more or less pleasure during the consumption of the reward (e.g., learning about theories on the origin of the universe might be an awe-inducing experience whereas learning the details of how big fast-food chains produce their burgers might be a very repulsive experience). The final phase of this epistemic reward cycle would be learning, both through conscious (e.g., learning the acquired knowledge) and unconscious processes (e.g., adjusting the probability of ever wanting to know more about a specific subject again)

The reward-anticipation account is mainly grounded on neuroscientific findings, especially works using fMRI (e.g., Kang, 2009; Gruber et al., 2014). Also called the “information-as-reward hypothesis” (Marvin & Shohamy, 2016), this line of thought posits that “curiosity conforms to basic characteristics of reward-motivated behavior” (Marvin & Shohamy, 2016, p. 266). According to this approach, interesting knowledge is rewarding per se (Murayama et al., 2019) and curiosity is the motivation to obtain that epistemic reward (Marvin & Shohamy, 2016; see Fig. 2). Once knowledge is obtained, just as in other reward-learning instances with typical rewards such as food or money, the organism compares the expected reward with the actual one felt, and updates future expectations (Marvin & Shohamy, 2016). Overall, the information-as-reward hypothesis received support from fMRI (Gruber et al., 2014; Kang et al., 2009), EEG (Alicart et al., 2020; Rüterbories et al., 2024), physiological (Baranes et al., 2015; Kang et al., 2009) and behavioral studies (Wang, 2019; Wang & Huang, 2018; Wiggin et al., 2019).

The reward-anticipation account has been so compelling that Murayama and colleagues suggested that notions such as ‘curiosity’ and ‘interest’ could be left aside when focusing on human motivation for learning (especially because the definitions of these two notions elicit much debate, see Grossnickle, 2016): instead, these authors proposed a “reward-learning framework of autonomous knowledge acquisition” (Murayama et al., 2019, p.878). This framework is based on standard reward-learning models with a conditioned stimulus (i.e., the knowledge-gap), an action (i.e., the knowledge-seeking behavior) and an unconditioned stimulus, the reward (i.e., the acquisition of interesting knowledge; Murayama, 2022). The acquisition of knowledge is rewarding, might or might not exceed reward expectations, and might reinforce future knowledge-seeking behaviors (Murayama, 2022; Murayama et al., 2019). The acquired knowledge also contributes to the knowledge base of the individual, leading to more question generation, a new cycle of knowledge-seeking, and thus to sustainable learning (Murayama, 2022). Some factors such as environmental structure, coincidences, expectancy beliefs, perceived costs, personality traits, and emotional valence are mentioned as possible factors in the awareness of knowledge-gaps and knowledge-seeking behaviors, but no explicit mechanism of curiosity elicitation is provided.

Other frameworks do recognize reward-like components of curiosity (i.e., prediction errors, use of dopaminergic pathways, ventral striatum activation) but refrain from calling curiosity a reward anticipation process. This is the case for instance of the Prediction, Appraisal, Curiosity, and Exploration (PACE) framework proposed by Gruber and Ranganath (2019). This framework rather focuses on how curiosity enhances memory: it posits that curiosity is triggered by prediction errors (i.e., due to novelty or unexpectedness in the context) or information gaps (i.e., information-based prediction errors). Novelty or unexpectedness-based prediction errors are associated to the hippocampal activity, while cognitive conflict involves anterior cingulate cortex activity. Prediction errors and information gaps are appraised in the lateral prefrontal cortex, and trigger either curiosity, associated with ventral striatum and ventral tegmental area/substantia nigra, or anxiety, associated with amygdala activation. If curiosity is elicited, the individual engages in exploration and knowledge-seeking behaviors. When desired knowledge is acquired, curiosity enhances memory by its facilitatory effect on the hippocampal activity, during encoding. This memory facilitation happens for the acquired knowledge but also for incidental information encoded during the experience of curiosity (Gruber et al., 2014). A particularly innovative aspect of the PACE framework is to combine the “prediction-error” and the “information-gap” frameworks with the proposal that the appraisal framework should also be considered when explaining how curiosity is triggered. This model leaves room for further consideration concerning the exact appraisal processes that take place in curiosity elicitation. Noteworthy, this model does not fully commit to the idea that curiosity is a reward-anticipation, only that it shares a lot with an external reward anticipation process (for a detailed discussion, see Gruber & Ranganath, 2019).

## An Integrative Appraisal Model of Epistemic Curiosity

**Fig. 3:**
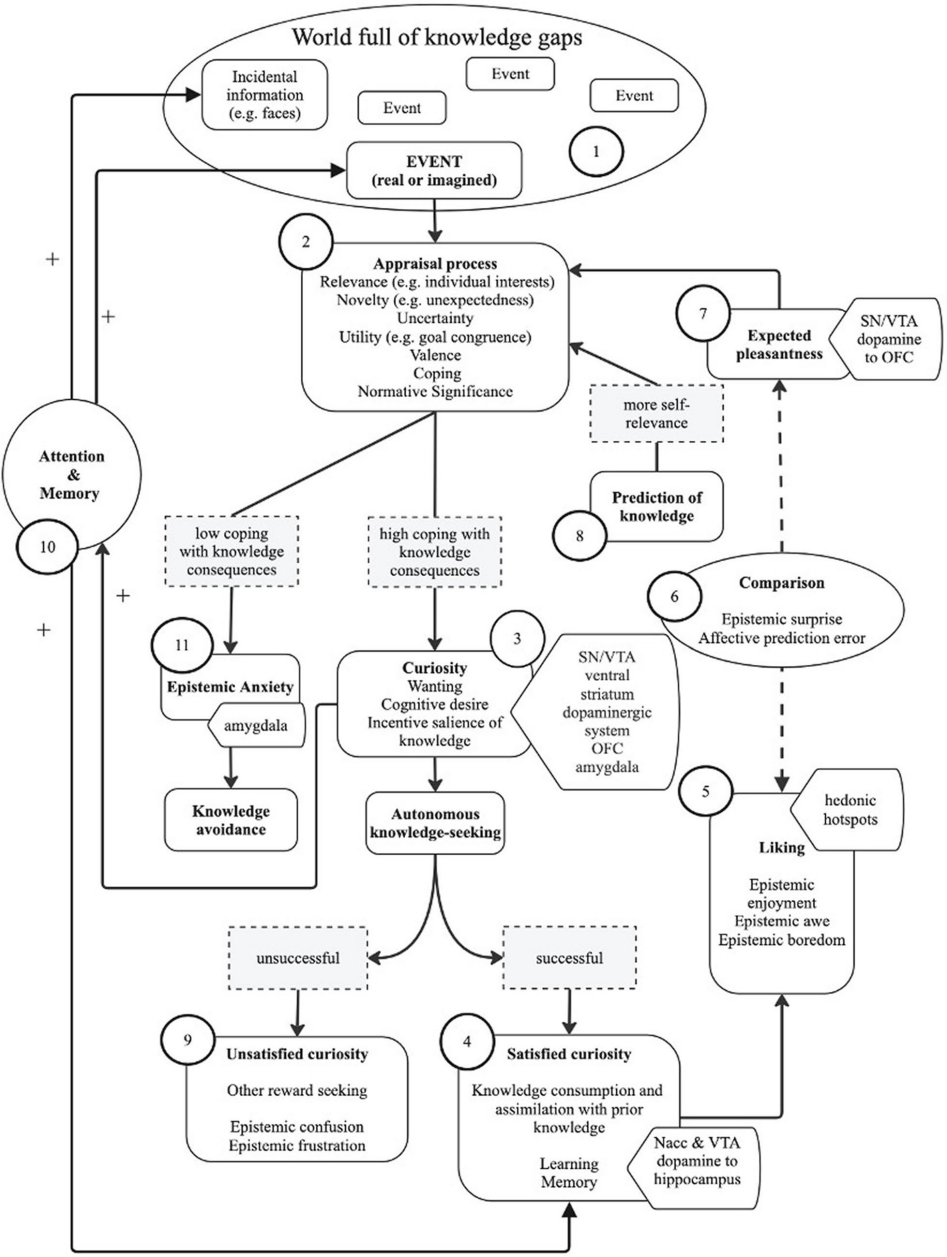
The integrative appraisal model of epistemic curiosity (IAMEC)

The existence of several models of curiosity, with a rich history and illuminating current research, is a sign of lively research on curiosity in affective science. We suggest that a large body of theoretical and empirical work on curiosity can be represented under one coherent model, the integrative appraisal model of epistemic curiosity (IAMEC, see Fig. 3). This theoretical proposition integrates information-gap (Loewenstein, 1994; Markey & Loewenstein, 2014), reward-anticipation related information seeking (FitzGibbon et al., 2020; Gruber & Ranganath, 2019; Murayama et al., 2019), and emotion-accounts of curiosity (Connelly, 2011; Silvia, 2005, 2008, 2010). Importantly, this model also takes into account theories of information-seeking (Sharot & Sunstein, 2020), appraisal theories of emotions in academic contexts (e.g., the control-value appraisal model proposed by Pekrun, see Pekrun, 2021), and more generally appraisal theories of emotions (Sander et al., 2005), as well as the epistemic emotion dynamics (D’Mello & Graesser, 2012; Muis et al., 2018; Pekrun et al., 2017). Here, we explicitly only focus on curiosity theories relevant for knowledge processing and acquisition, hence the use of the term “epistemic” curiosity. The integration of reward and emotion models of curiosity is also in convergence with links between emotion and reward (see Sander & Nummenmaa, 2021).

A key acceptation of this model is that the world is full of knowledge gaps (see point 1 in Fig. 3). A common challenge of previous curiosity theories (e.g., reward and information-gap approaches) is that they do not explain why some knowledge-gaps elicit curiosity while others are ignored. Indeed, if the detection of a knowledge gap was enough to elicit a desire to fill the gap, encyclopedias would be addictive. The IAMEC posits that knowledge gaps are subjected to automatic cognitive appraisals.

The IAMEC suggests that a knowledge gap triggers curiosity only when a specific subjective evaluation (i.e., a series of appraisals) of it is computed by the individual (Sander, 2013; Scherer & Moors, 2019; see point 2 in Fig. 3). Both the PACE framework (Gruber & Ranganath, 2019) and the emotional accounts of curiosity have highlighted the role of appraisal processes in the triggering of curiosity. The multicomponential appraisal theories of emotion (see Scherer & Moors, 2019; see Sander et al., 2018) are particularly well suited for the specification of the appraisal process that elicits curiosity. Indeed, previous studies found that novelty (Silvia, 2005), pleasantness (Marvin & Shohamy, 2016; van Lieshout et al., 2018, 2021), unexpectedness (Vogl et al., 2020), concern relevance (e.g., individual interest relevance or value see Pekrun, 2021; Dubey & Griffiths, 2020; Horn et al., 2024; Spitzer et al., 2024), uncertainty (van Lieshout et al., 2018, 2021; Vogl et al., 2020; especially moderate uncertainty in Kang et al., 2009; Dubey & Griffiths, 2020; Spitzer et al., 2024) and coping potential (Noordewier & Goclowska, 2024; Silvia, 2005; also called control in Pekrun, 2021) are all appraisal processes that are positive predictors of curiosity. Goal-relevance, as well as goal-conduciveness, are also proposed to be positive predictors of curiosity even though in such cases it might be argued that knowledge is no longer non-instrumental (Connelly, 2011; see Dubey et al., 2022 for detailed discussion). Importantly, unpleasantness (e.g., morbid knowledge: see Oosterwijk, 2017; Oosterwijk et al., 2020, and regret-inducing knowledge: see FitzGibbon et al., 2021) and norm violating knowledge (e.g., gossip or true-crime related knowledge) can also trigger curiosity as long as the individual considers that they can handle the knowledge in question and adjust to the situation (i.e., they appraise the knowledge with a high coping potential). For example, one might want to find out about their spouse’s infidelity, if they feel like that knowledge will allow them to end the relationship or repair the relationship. If divorce is impossible because of economic difficulties, one might fear finding out about an affair and rather avoid such knowledge.

**Fig. 4:**
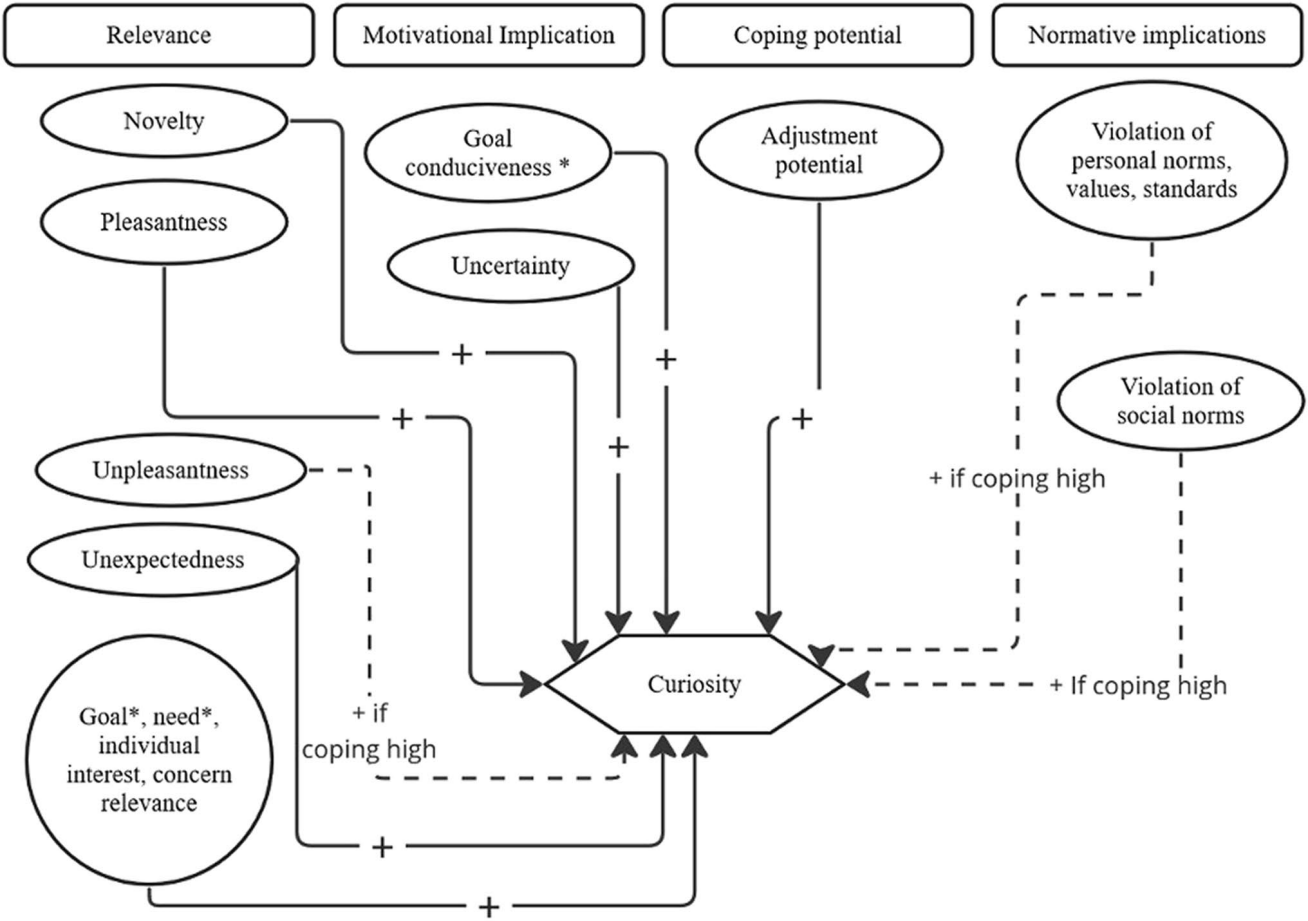
The suggested appraisal structure of curiosity, based on the Component Process Model. *Note*: Full lines are positive predictors of curiosity. Discontinued lines are positive predictors only if perceived coping potential is high. To be more specific, high novelty, high pleasantness, high unexpectedness, high uncertainty, high adjustment potential to the information are all positive predictors of curiosity. Unpleasantness, violation of individual standards, and violation of social standards are positive predictors of curiosity only if perceived coping potential is high (i.e., the individual can handle the information). Otherwise, information may cause anxiety instead of curiosity. “*” means that information is no longer non-instrumental

Findings on the predictors of curiosity are abundant, and we found the Component Process Model proposed by Scherer (Grandjean et al., 2008; Sander et al., 2005; Scherer, 2001) to be a useful guide to model the predictors (see Fig. 4 for the suggested appraisal profile of curiosity, inspired by the Component Process Model). This model classifies appraisals into four categories: relevance (e.g., significance for well-being or the self), motivational implication (e.g., consequences for immediate or long-term goals), coping potential (e.g., capacity to cope with or to adjust to the consequences of the evaluated object) and normative implications (e.g., compatibility of the event with respect to personal values and social norms). Each of these major categories have subsets of stimulus evaluation checks: relevance detection refers to the evaluation of novelty (which can itself be subdivided into the features of suddenness, familiarity and predictability, see Sander et al., 2005), intrinsic pleasantness and goal/need relevance (Sander et al., 2005; Scherer, 2001). Motivational implication assessment refers to the evaluation in terms of causal attribution (i.e., is the event attributable to a person and if so to whom?), outcome probability (i.e., the certainty around consequences), expectedness, goal or need conduciveness, and finally, urgency (Sander et al., 2005; Scherer, 2001). The determination of coping potential refers to the evaluation of control, power, and potential for adjustment (Sander et al., 2005; Scherer, 2001). Finally, the normative implications evaluation refers to the evaluation of the object against internal norms (e.g., personal values, internalized cultural norms) and external norms (e.g., societal or institutional norms).

When curiosity is triggered (see point 3 of Fig. 3), mechanisms such as the conscious and unconscious wanting of rewards with incentive salience and cognitive desire may take place (FitzGibbon et al., 2020). The individual experiences higher arousal, an approach motivation tendency, a bodily expression of curiosity, and a conscious feeling of curiosity (e.g., Dukes et al., 2017 for the bodily expression of interest and Kang et al., 2009 for pupil dilation). Neuroimaging studies suggest that a state of curiosity is characterized by the enhanced dopaminergic activity of SN/VTA neurons and OFC region (Charpentier et al., 2018; Gruber et al., 2014; Kang et al., 2009; Lau et al., 2020; Ligneul et al., 2018; Poh et al., 2022). Whether curiosity is a reward anticipation or an emotion may well be a question primarily raised by differences between research traditions than an empirical question as such. Indeed, different theoretical perspectives, originating from distinct research traditions, may bring complementary approaches that are not mutually exclusive to the very same phenomenon. With this respect, curiosity is proposed to be both an emotion and a reward anticipation process, in line with other theoretical work suggesting a conceptual overlap between the emotional and motivational processes (Sander & Nummenmaa, 2021).

It should be noted that, in the IAMEC, curiosity is associated with increased motivation for knowledge-seeking but whether knowledge-seeking actually happens as a behavior is probably at least partially dependent on situational aspects (e.g., a funeral is probably a bad place to google information on your phone). If knowledge-seeking is successful (i.e., the object of the individual’s curiosity is obtained), then curiosity is satisfied, knowledge consumption and knowledge assimilation occur: new knowledge is integrated with the prior knowledge through memory consolidation and learning mechanisms (see point 4; see Murayama, 2022). This mechanism is triggered by the dopaminergic activity supposed to be projected from the nucleus accumbens to the hippocampus (Gruber et al., 2014).

Conceptualizing curiosity as a reward anticipation state allows to consider the emotional experience of knowledge consumption as conceptually similar to the ‘liking’ component of reward processing (Berridge, 1996; see point 5). This experience is probably linked to the activity of ‘hedonic hotspots’ (see Morales & Berridge, 2020 for details on hotspots) such as the nucleus accumbens “shell”, ventral pallidum and OFC (see Pool et al., 2022). Relying for instance on reward prediction mechanisms, in epistemic emotion terms, a person might feel epistemic enjoyment, epistemic interest (e.g., post-answer interest in Fastrich et al., 2018; Fandakova & Gruber, 2021), epistemic awe, or even epistemic disappointment, or epistemic boredom during this “liking” phase.

Once knowledge is consumed and the individual experiences ‘liking’, the hedonic experience of the true ‘liking’ phase is compared to the expected pleasure (see point 6). This is what some researchers focus on as the ‘information prediction error’ (Marvin & Shohamy, 2016; see also affective prediction errors in Vollberg & Sander, 2024). Usually, the information prediction error is measured, in this context, as the difference of the satisfaction felt after acquiring the knowledge (e.g., seeing the answer of the trivia question) with the curiosity felt before getting the knowledge. Typically, this is supposed to elicit surprise and is thought to imply additional positive influence on memory (Ligneul et al., 2018).

In congruence with reward-learning frameworks of curiosity (FitzGibbon et al., 2020; Murayama et al., 2019), the comparison of true ‘liking’ with expected pleasantness is thought to update future expected pleasantness (see point 7). The updating of expected pleasantness is thought to be supported by midbrain dopaminergic projections to OFC (Howard & Kahnt, 2018).

The repetition of curiosity and enjoyment/interest experiences when interacting with a particular topic (e.g., football or painting) enhances the expected pleasantness of knowledge-seeking outcomes related to the topic and is thought to add to the development of individual interests, as suggested by the four-phase interest development model (Hidi & Renninger, 2006).

Previous research showed that if people are prompted to make a prediction about the knowledge (see point 8 of Fig. 3), curiosity is enhanced (Brod & Breitwieser, 2019). This may be because making a prediction about the not-yet-acquired knowledge adds more self-relevance to the appraisal process (i.e., “was I right or wrong in my guess?”), thus enhancing curiosity. Similarly, making a choice between two lotteries enhanced self-reported curiosity (Romero Verdugo et al., 2023). Such results are hard to explain without taking into account the appraisal of relevance.

In cases where one cannot access the curiosity-eliciting knowledge (e.g., lack of resources, lack of scientific discoveries on the subject, curiosity-eliciting adds, cliffhangers in movies with sequels) curiosity may remain unsatisfied (see point 10). Some studies have shown that unsatisfied curiosity elicits other reward-seeking (e.g., food) behaviors (Wang & Huang, 2018; Wang et al., 2019; Wiggin et al., 2019). In settings where knowledge is the focus and other rewards are unavailable (e.g., in a classroom), the individual might feel epistemic confusion and epistemic frustration.

Curiosity enhances attention (Baranes et al., 2015) and memory (Brod & Breitwieser, 2019; Duan et al., 2020; Fastrich et al., 2018; Galli et al., 2018; Gruber et al., 2014; Kang et al., 2009; Ligneul et al., 2018; Marvin & Shohamy, 2016; Stare et al., 2018; see point 10) which have a positive impact on knowledge acquisition and assimilation (point 4) and even enhances memory for incidental stimuli (Gruber et al., 2014, though this enhancement might be limited, see Keller et al., 2024).

Sometimes knowledge gaps cause anxiety (point 11 of Fig. 3). Perceived coping potential is key in the elicitation of anxiety instead of curiosity (see point 11). Amygdala is thought to be a key region linked to both positive and negative emotions because of its key role in the appraisal of concern-relevance (see Cunningham & Brosch, 2012; Sander et al., 2003; Murray et al., 2023) that could lead either to anxiety and knowledge-avoidance or to curiosity and knowledge-seeking.

## Future Directions

The model presented in this paper is aiming at enhancing cohesive interdisciplinary collaboration in the study of curiosity by combining the information-gap, emotion and reward-anticipation accounts of curiosity, showing the key compatibilities between these approaches (without denying differences between the approaches). In particular, including appraisal processes at the heart of the model brings additional value to prior theories by accounting for why not all topics with knowledge gaps elicit curiosity, why predictions about knowledge enhance curiosity, takes into account unsatisfied curiosity, and provides testable hypotheses for the appraisal profile of curiosity.

This model can be tested by manipulating the appraisals of the knowledge gap, as they are defined in the Component Process Model (see Fig. 4 for exact predictions). The key claim of the IAMEC regarding the elicitation phase is that curiosity-eliciting non-instrumental knowledge gaps have a specific appraisal profile mostly characterized by high relevance and high coping potential, based on prior findings (see point 2 of Fig. 3 and Fig. 4). To falsify this model, it would suffice to find cases where epistemic curiosity is elicited by low relevance and low coping potential knowledge gaps. Further, the model can also be falsified by showing that individuals avoid future exploration of topics with prior positive affective prediction error (i.e., cases where the actual liking of filling the knowledge gap is higher than expected pleasantness). For instance, two-armed bandits’ tasks might be adapted to create topics with positive or negative affective prediction errors, using validated trivia questions datasets (see Fastrich et al., 2018 for a database).

Our psychological and neuroscientific account may be complemented by computational accounts of epistemic curiosity (Dubey & Griffiths, 2020; Gottlieb & Oudeyer, 2018; Poli et al., 2024). Two main approaches are dominant in this literature to explain curiosity-driven behavior: learning progress hypothesis (Oudeyer et al., 2016; Poli et al., 2022) and active inference perspective (Friston et al., 2015, 2017; Kiverstein et al., 2019; Schwartenbeck et al., 2013, 2019; Ueltzhöffer, 2018). Such accounts of curiosity are beneficial for synthetic agent performance (Dubey & Griffiths, 2020), and may also benefit to a computational modeling of IAMEC. Finally, it is yet unclear how this model for epistemic curiosity applies to other types of curiosity: future developments should focus on testing this model’s application to, for instance, perceptual curiosity, social curiosity and morbid curiosity.

## Conclusion

In this paper we aimed at presenting an integration of the multiple modern models of curiosity. More specifically, we provided an overview of old and current conceptualizations of curiosity and synthesized them in one coherent model, the integrative appraisal model of epistemic curiosity, which accounts for an extensive scope of findings in curiosity research. The integrative appraisal model of curiosity posits that epistemic curiosity is elicited by a specific pattern of appraisal processes, relies on the reward circuit, functions as a reward anticipation mechanism, can be conceptually related to the reward components of wanting and liking, and enhances attention, memory and learning. The nature of curiosity seems congruent with the very close interplay of reward and emotion (Emanuel & Eldar, 2023; Sander & Nummenmaa, 2021; Vollberg & Sander, 2024) and we believe this framework only adds to integrative efforts in the affective sciences.

## Additional Information

### Availability of data and material (data transparency)

No datasets were generated or analysed during the current study.
